# The Cardiovascular-Mortality-Based Estimate for Normal Range of the Ankle–Brachial Index (ABI)

**DOI:** 10.3390/jcdd9050147

**Published:** 2022-05-05

**Authors:** Essi Peltonen, Mirjami Laivuori, Damir Vakhitov, Päivi Korhonen, Maarit Venermo, Harri Hakovirta

**Affiliations:** 1 Department of Surgery, University of Turku, 20500 Turku, Finland; essi.peltonen@tyks.fi; 2 Department of Vascular Surgery, Abdominal Center, Helsinki University Hospital and University of Helsinki, 00100 Helsinki, Finland; mirjami.laivuori@hus.fi (M.L.); maarit.venermo@hus.fi (M.V.); 3 Centre for Vascular Surgery and Interventional Radiology, Tampere University Hospital, 33520 Tampere, Finland; damir.vakhitov@fimnet.fi; 4 Department of General Practice, University of Turku, 20500 Turku, Finland; paikor@utu.fi; 5 Department of Surgery, Satasairaala, 28500 Pori, Finland

**Keywords:** ABI, TBI, cardiovascular, survival, mortality

## Abstract

Background: The ankle–brachial index (ABI) is a first-line examination in cardiovascular risk evaluation. Since cut-off values for normal ABI vary, the aim of the present study was to identify the cardiovascular-mortality-based estimate for the normal range of the ABI. After determining the reference range for the ABI, the corresponding toe–brachial index (TBI) and toe pressure for normal ABI were analyzed. Methods: All consecutive non-invasive pressure measurements in the vascular laboratory of a large university hospital 2011–2013 inclusive were collected and combined with patient characteristics and official dates and causes of death. Patients with an ABI range of 0.8–1.4 on both lower limbs were included in this study. Results: From 2751 patients, 868 had bilateral ABI values within the inclusion. Both ABI category ranges 0.80–0.89 and 0.90–0.99 had poorer survival compared to ABI categories 1.00–1.29 (*p* < 0.05). The 1-, 3-, and 5-year cardiovascular-death-free survival for respective ABI categories 0.80–0.99 vs. 1.00–1.29 were 90% vs. 96%, 84% vs. 92%, and 60% vs. 87%. The 1-, 3-, and 5-year overall survival for ABI categories 0.80–0.99 vs. 1.00–1.29 were 85% vs. 92%, 75% vs. 83%, and 42% vs. 74%. Conclusions: Borderline ABI (0.90–0.99) associates with higher overall and cardiovascular mortality compared to ABI values 1.00–1.29.

## 1. Introduction

Cardiovascular morbidity is a significant cause of functional disability and mortality [1]. Peripheral artery disease (PAD) is the strongest risk factor for major adverse cardiovascular events (MACE) and cardiovascular-associated mortality [1,2]. The ankle–brachial index (ABI) is the ratio between the ankle and brachial systolic blood pressures (SPB) and is a simple, non-invasive and inexpensive method to detect PAD that is also used to identify patients with high cardiovascular risk. The associations of abnormal ABI with the risk of cardiovascular events and death have been intensively studied. Nevertheless, there is a lack of consensus about normal ABI values, as these vary between 0.90 and 1.4 [2,3,4,5,6].

The lower limit for normal ABI is considered to be between 0.90 and 1.10 [7,8,9,10,11,12,13]. Previous studies suggest the lower cut-off level at 0.90 [2,5,6,14,15]. Several studies and consensus statements, however, propose a borderline range of 0.90–0.99, below which ABI is associated with an increased risk of cardiovascular morbidity and mortality [7,15,16,17,18]. Therefore, the lower cut-off value for normal ABI remains controversial.

A strong body of evidence concerning ABI and cardiovascular risk has hitherto omitted data for abnormally high ABI values [7,19]. Nonetheless, it is well established that the stiffening of the arterial wall and abnormally high ABI values are associated with an increased risk for cardiovascular events and even higher mortality than those values that are below normal [3,9,10,12]. According to the literature, the upper ABI cut-off for abnormal high ABI varies between 1.3 and 1.4 [3,9].

The aim of the present study was to identify the lower and upper cut-off values for normal ABI based on the cardiovascular mortality of the selected cohort.

## 2. Materials and Methods

The present study is a retrospective registry-based cohort study. The data consist of all consecutive ABI measurements performed in the vascular laboratory at the department of clinical physiology of the University Hospital of Turku from 1 January 2011 to 31 December 2013. All patients had leg-related symptoms, and ABI measurements were the first line of investigation before vascular consultation. The vascular laboratory serves as a single unit and provides standardized non-invasive pressure measurements that cover a population of 480,000 inhabitants. All non-emergent patients referred to vascular surgical outpatient treatment had ABI and toe–brachial index (TBI) measurements. Only patients with bilateral ABIs of 0.80–1.40 were included in the group since these individuals were considered to present normal or borderline ABI values and a low risk for cardiovascular and overall mortality. In the case of multiple measurements, the first measurement during the study period for each patient was recorded. Patient files were followed-up until 1 January 2020, which was considered the end of the study. The review board of the University of Turku reviewed and accepted the study (IRB number T344/2017). Due to the nature of the study, no informed consent from the patients was required.

### 2.1. Non-Invasive Pressure Measurements

Trained nurses took ABI, TBI, and toe pressure (TP) measurements in accordance with European Society for Vascular Surgery guidelines [2]. All measurements were performed with the patient in the supine position with the feet at heart level and in a standardized environment. The protocol entails the systolic arterial pressure of the anterior tibial and posterior tibial artery blood pressures being measured at the level of the ankle; for TP, it was measured at the big toe, and the systolic pressure of the brachial artery with measured with the laser Doppler method using a Periflux 6000 (Perimed AB, Järfälla, Sweden) device. When possible, non-invasive pressures were measured for both lower limbs. ABI was calculated by dividing the higher ankle SBP with the corresponding higher brachial SBP [3]. Only patients with ABI values between 0.80 and 1.40 on both limbs were included in the study cohort. The ABI of the limb with lower value was set as the index ABI. For the initial analyses, ABI indices were divided into 6 category ranges: 0.80–0.89, 0.90–0.99, 1.00–1.09, 1.10–1.19, 1.20–1.29, and 1.30–1.40.

### 2.2. Data Collection

Baseline characteristics and medical history of the patients were collected retrospectively from the Hospital District of Southwest Finland electronic patient registry, which covers all operation and patient records in the hospital district. Comorbidities were listed for each patient in accordance with the Finnish version of the ICD-10 (International Statistical Classification of Diseases and Related Health Problems). The causes and dates of death were provided by the Causes of Death Registry of Statistics Finland. The Causes of Death Registry registers all deaths of people with permanent residency in Finland. The registry comprises comprehensive data on mortality in Finland. Only 0.2% of all causes of death remain unspecified annually [20].

### 2.3. Statistical Analysis

All statistical analyses were performed using SPSS version 27 for (IBM, Armonk, NY, USA). Descriptive statistics were used to compare patient demographics. Mean values and standard deviation (SD) were applied for normal distributions. The Fisher’s exact test was applied to compare categorical variables, and the Student’s *t*-test and ANOVA were used to compare continuous variables after normal distributions of the values were tested using the Shapiro–Wilk test. Survival was calculated by Kaplan–Meier survival analysis, and risk analyses were conducted using multinominal logistic analyses. According to the power analysis for survival analyses, the sample size was estimated at 99 for each range category. *p*-values ≤0.05 were considered statistically significant.

## 3. Results

### 3.1. Study Cohort and Demography

**Figure 1:**
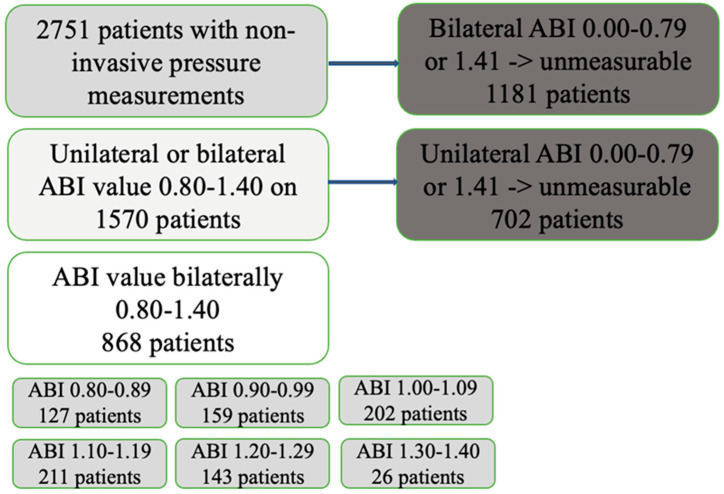
Flow chart of the study. Patients with bilateral ankle–brachial index (ABI) 0.80–1.40 were included in the initial survival analysis.

A total of 2751 patients had non-invasive lower limb pressure measurements at the department of clinical physiology at Turku University Hospital during the study period. Of these, 868 had bilateral ABI values between 0.80 and 1.40 and were included in the study. Figure 1 shows the study flow chart, and Table 1 and Table 2 show the demography.

### 3.2. ABI Range Category and Survival

The estimated survivals and cardiovascular-death-free survivals and 1-, 3-, and 5-year survival rates for each ABI range category are presented in Table 3A,B.

The associations between age-adjusted overall mortality and ABI range category were assessed by multinominal logistic analyses. The ABI range category of 0.80–0.89 was set as the reference. Odds Ratio (OR) values for the ABI range categories were as follows: 0.90–0.99 OR = 0.949 (*p* = 0.841), 1.00–1.09 OR = 0.411 (*p* < 0.001), 1.10–1.19 OR = 0.526 (*p* < 0.01), 1.20–1.29 OR = 0.452 (*p* = 0.004), and 1.30–1.40 OR = 0.975 (*p* = 0.071). Correspondingly, the ORs for age-adjusted overall mortality for the ABI range category of 0.90–0.99 set as reference were: 0.80–0.89 OR = 1.05 (*p* = 0.841), 1.00–1.09 OR = 0.433 (*p* < 0.001), 1.10–1.19 OR = 0.554 (*p* = 0.011), 1.20–1.29 OR = 0.476 (*p* = 0.005), and 1.30–1.40 OR = 0.601 (*p* = 0.283)

Similarly, associations between age-adjusted cardiovascular mortality and the ABI range category were assessed. The ORs for the the ABI range category of 0.80–0.89 set as reference were: 0.90–0.99 OR = 0.856 (*p* = 0.579), 1.00–1.09 OR = 0.442 (*p* = 0.004), 1.10–1.19 OR = 0.348 (*p* < 0.001), 1.20–1.29 OR = 0.449 (*p* = 0.014), and 1.30–1.40 OR = 0.383 (*p* = 0.147). Correspondingly, the ORs for age-adjusted cardiovascular mortality for the ABI range category of 0.90–0.99 set as reference were: 0.80–0.89 OR = 1.17 (*p* = 0.579), 1.00–1.09 OR = 0.516 (*p* = 0.017), 1.10–1.19 OR = 0.406 (*p* = 0.002), 1.20–1.29 OR = 0.525 (*p* = 0.042), and 1.30–1.40 OR = 0.447 (*p* = 0.221)

**Figure 2:**
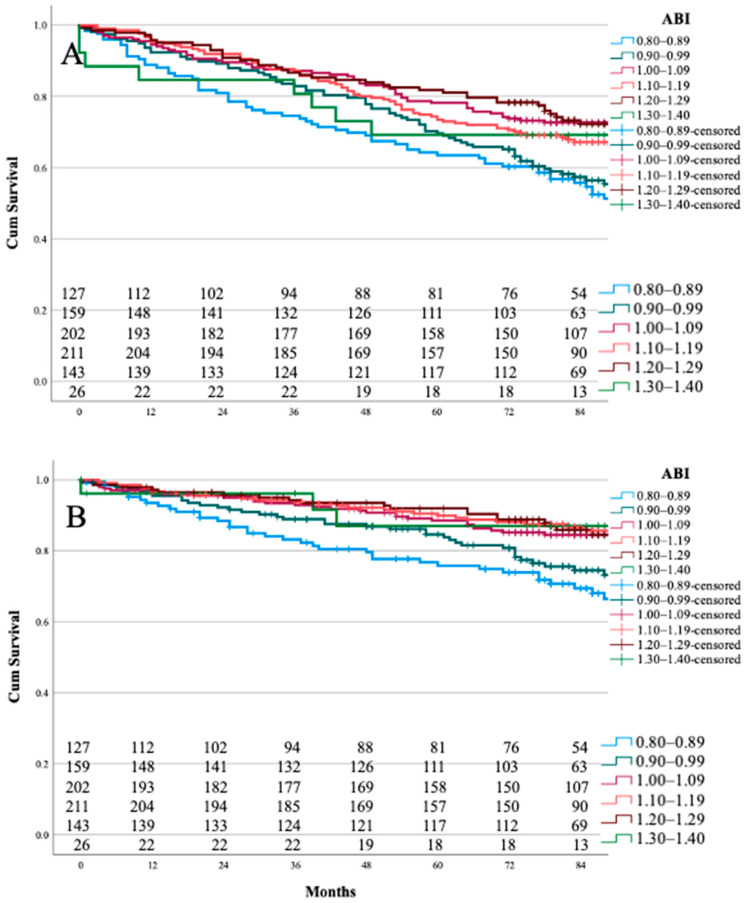
A-B Kaplan–Meier curves for 8-year survival for ABI 0.80–1.40. Panel (**A**), overall survival; (**B**), cardiovascular-death-free survival.

The Kaplan–Meier curves for both overall and cardiovascular-death-free survivals are presented in Figure 2A,B.

### 3.3. TBI and Toe Pressure Values

The corresponding TBI and toe pressure values for patients with the highest cardiovascular-death-free survival (ABI 1.00–1.29) were calculated. A total of 582 patients with bilateral ABI (1.00–1.29) were identified. The mean TP for these patients was 97.1 mmHg (SD 25.7), and corresponding TBI values had a mean of 0.711 (SD 0.173).

## 4. Discussion

According to our present observations, the cardiovascular-death-free and overall survival for patients with ABI values between 1.00 and 1.29 are better compared to the ABI range of 0.80–0.90. The borderline ABI values of 0.90–0.99 are associated with increased mortality compared to the ABI range category of 1.00–1.29. Another non-invasive index TBI was measured based on these ABI values. The corresponding TBI for ABI values 1.00–1.29 was 0.711 (SD 0.173).

### 4.1. Normal Range for ABI

Although ABI is a potent tool for cardiovascular risk analyses, the normal lower cut-off value varies between 0.90 and 1.10 [7,13,18,19]. A majority of the literature and international guidelines state an ABI of 0.90 as the cut-off for a two-fold increased risk of a cardiovascular event [2,5,6]. Recently, borderline ABI has become accepted as a concept that refers to values between 0.90 and 0.99. Many cohort studies suggest that borderline ABI seems to be associated with increased cardiovascular morbidity and mortality [15,16,17,21] in comparison to the normal ABI range of 1.00–1.40. An IMPACT-ABI study on symptomatic peripheral arterial disease (PAD) patients demonstrated that even a 10% higher MACE at 4 years and cardiovascular death [17] among the borderline ABI group compared to ABI range category 1.00–1.40 is in line with the findings of an ARTPER cohort study with a 9-year follow-up. Borderline ABI patients are exposed to a higher incidence of MACE annually in comparison to those with normal ABIs (1.00–1.40) [21,22]. According to the present study and data obtained from other studies, the normal lower cut-off value for ABI is 1.00, and our data also demonstrate a 10% higher cardiovascular mortality rate for the borderline ABI category (0.90–0.99). Interestingly we did not detect significant differences between ABI ranges 0.80–0.89 and 0.90–0.99, although the survival curves showed small differences between categories.

The upper ABI cut-off value in cardiovascular risk evaluation is accepted as 1.30–1.40 [5,6,13,15,19,23,24]. A study that investigated the relationship between an elevated ABI and the presence of medial arterial calcification (MAC) containing type II diabetic patients demonstrated a test specificity of 15.7% and 30.9%, with a sensitivity of 93.6% and 83.6% for the corresponding cut-off values ≥1.40 and ≥1.30 [25]. In the present study, the approach to detecting the upper cut-off value was different. The cardiovascular-death-free and overall survival rates were compared between categories for every 0.1 increase for ABI values 0.80–1.40. The log-rank test, age-adjusted multilogistic binary analyses, and survival curves all supported similar survival for the ABI categories of 1.00–1.09, 1.10–1.19, and 1.20–1.29. Unfortunately, category 1.30–1.40 was underpowered, as it only contained 26 patients, but by using Kaplan–Meier curve analysis, we found the trend for cardiovascular-death-free survival was similar to that of ABI values of 1.00–1.29. In addition, the ratio of cardiovascular cause of death for ABI values 1.30–1.40 corresponded to the above-mentioned normal ABI values.

### 4.2. Normal TBI

Carter and Lezack suggested that normal values for TBI vary depending on the comorbidities [26]. The normal values that those authors found were as follows: “young normal” 0.86 ± 0.03, “old normal” 0.91 ± 0.04, patients with diabetes mellitus (DM) 0.83 ± 0.04, coronary artery disease 0.87 ± 0.04, and hypertension 0.81 ± 0.03 [26]. Later, the cut-off value for TBI recommended for patients with DM and abnormally high ABI (>1.40) was 0.70 for TBI [4,27]. The study by Hyun et al. set the range of normal TBI to 0.62–1.08 [13], which they suggested might have diluted the predictive value of TBI in their report. We measured the mean and median TBI for patients with the estimated normal ABI (1.00–1.40) to identify the normal TBI for the present cohort. Based on the present study’s data, the TBI corresponding to normal ABI was 0.711 (SD 0.173), which supports the normal TBI value found in recent guidelines [4]. However, the 0.10 discrepancy between normal values can be explained by a different approach compared to that used in the pioneering work of Carter and Lezack [4,26,27]. Recent guidelines and studies focus on the cut-off for a cardiovascular event and do not determine the normal value in subjects with no detectable PAD. To our knowledge, there is no detailed published analysis of cardiovascular and overall mortality of MACE, but the present study is based on categories with a relatively wide range of TBI values. Further studies are warranted to not only investigate the TBI value that is associated with a normal cardiovascular risk but also for patients without PAD who present with various comorbidities, including DM and coronary artery disease.

### 4.3. Normal TP

TP is widely used for detecting critical limb-threatening ischemia and malperfusion of the leg [5,6]. However, the value for normal toe pressure published in the literature is not reliable. Based on the present data for ABI, between 1.00 and 1.29, the mean toe pressure is 97.1 mmHg (SD 25.7).

### 4.4. Strengths and Limitations

Although the present study is based on a relatively large original cohort of patients, unfortunately, the anticipated number of patients in the range category ABI 1.30–1.40 has insufficient statistical power. The non-invasive pressure values were measured in a high-volume standardized vascular laboratory. Values were collected retrospectively from an electronic database as PDF files of the original datasheets. Due to the retrospective nature of our study, all demographic data were based on the official diagnosis. The accuracy was fair, but diagnoses such as atrial fibrillation might vary if treated and would be more reliably evaluated in an observational, prospective follow-up study.

## 5. Conclusions

Our present study data suggest that an ABI value of 1.00–1.29 is associated with a lower risk of cardiovascular mortality than ABI values < 1.00. The corresponding TBI for the ABI 1.00–1.29 is 0.711 (SD 0.173) and a toe pressure value of 97.1 mmHg (SD 25.7).

## 6. Patients

The present sample contains unselected patients with leg-related symptoms. Hospital policy requested non-invasive pressure measurements before considering elective vascular consultation. Therefore, the cohort contains a high number of patients with bilateral normal ABI values.

## Figures and Tables

**Table 1:** Demography for study cohort of 868 patients with ABI 0.80–1.40.

	ABI 0.80–1.40 (*n* = 868)
	mean (SD)
Age	67.9
	*n* (%)
Sex male	505 (58.2)
CAD	236 (27.2)
CHF	148 (17.1)
HT	466 (53.7)
FA	204 (23.5)
CeVD	103 (11.9)
DM	297 (34.2)
DMI	58 (6.7)
DMII	239 (27.6)
Dyslipidaemia	195 (22.5)
CKD	106 (12.2)
COPD	87 (10.0)
Rheumatoid disease	61 (7.0)
Varicose Ulcer	97 (11.2)
CV death	160 (18.4)

CAD = coronary artery disease, CHF = chronic heart failure, CKD = chronic kidney dysfunction, COPD = chronic obstructive pulmonary disease, CV = cardiovascular, CeVD = cerebrovascular disease, DM = diabetes mellitus, DM = diabetes mellitus, DM I = type I diabetes mellitus, DM II = type II diabetes mellitus, FA = atrial fibrillation, HT = hypertension, SE standard error.

**Table 2:** Demography for ABI range categories 0.80–0.89, 0.90–0.99, 1.00–1.09, 1.10–1.19, 1.20–1.29, and 1.30–1.40.

				ABI Range Categories			
	0.80–0.89	0.90–0.99	1.00–1.09	1.10–1.19	1.20–1.29	1.30–1.40	*p* value
			Mean	(SD)			
Age	70.8 (11.8)	68.9 (15.2)	68.11 (13.1)	68.38 (13.5)	64.2 (16.5)	63.9 (15.0)	<0.001
			*n*	(%)			
Sex male	79 (62.29	85 (53.5)	110 (54.5)	120 (56.9)	91 (63.6)	20 (76.9)	0.105
CAD	42 (33.1)	57 (35.8)	60 (29.7)	48 (22.7)	21 (14.7)	8 (30.8)	<0.001
CHF	32 (25.2)	31 (19.5)	28 (13.9)	33 (15.6)	18 (12.6)	6 (23.1)	0.053
HT	68 (53.5)	89 (56.0)	111 (55.0)	120 (56.9)	69 (48.3)	9 (34.6)	0.238
FA	32 (25.2)	36 (22.6)	47 (23.3)	53 (25.1)	29 (20.3)	7 (26.9)	0.893
CeVD	16 (12.6)	21 (13.2)	21 (10.4)	23 (10.9)	18 (12.6)	4 (15.4)	0.904
DM	43 (33.9)	63 (39.6)	55 (27.2)	68 (32.2)	54 (37.8)	14 (53.8)	0.034
DMI	7 (5.5)	12 (7.5)	14 (6.9)	13 (6.2)	9 (6.3)	3 (11.5)	0.867
DMII	37 (29.1)	50 (31.4)	42 (20.8)	55 (26.1)	45 (31.5)	11 (42.3)	0.065
Dyslipidaemia	35 (27.8)	44 (27.7)	40 (19.8)	38 (18.0)	36 (25.2)	2 (7.7)	0.039
CKD	19 (15.0)	21 (13.2)	22 (10.9)	26 (12.3)	14 (9.8)	4 (15.4)	0.759
COPD	24 (18.9)	22 (13.8)	18 (8.9)	14 (6.6)	6 (4.2)	3 (11.3)	<0.001
Rheumatoid disease	5 (3.9)	9 (5.7)	17 (8.4)	13 (6.2)	14 (9.8)	3 (11.5)	0.302
Varicose Ulcer	11 (8.7)	14 (8.8)	31 (15.3)	26 (12.3)	13 (9.1)	2 (7.7)	0.312
CV death	39 (30.7)	41 (25.8)	31 (15.3)	27 (12.8)	19 (13.3)	3 (11.5)	<0.001

CAD = coronary artery disease, CHF = chronic heart failure, CKD = chronic kidney dysfunction, COPD = chronic obstructive pulmonary disease, CV = cardiovascular, CeVD = cerebrovascular disease, DM = diabetes mellitus, DM I = type I diabetes mellitus, DM II = type II diabetes mellitus, FA = atrial fibrillation, HT = hypertension, SE standard error, *p* value for age AVOVA test, for categorical variables Fisher´s exact test.

**Table 3:** Survival and ABI range categories. The estimated overall survival and cardiovascular-death-free survivals in months (SE) of the ABI range categories are presented in Table 3A. Table 3B shows 1-, 3-, and 5-year survival rates for each category.

**A**	**Mean Survival**			**Mean CVDFS**		
**ABI**	**Months (SE)**	***p*** ** Value**	***p*** ** Value**	**Months (SE)**	***p*** ** Value**	***p*** ** Value**
0.80–0.89	73.0 (3.47)	reference	0.393	84.2 (3.25)	reference	0.264
0.90–0.99	78.8 (2.76)	0.393	reference	90.0 (2.49)	0.264	reference
1.00–1.09	87.8 (2.36)	<0.001	<0.001	96.2 (1.91)	<0.001	0.013
1.10–1.19	85.1 (2.28)	0.003	0.030	97.6 (1.77)	<0.001	0.003
1.20–1.29	89.4 (2.65)	<0.001	<0.001	98.0 (2.06)	<0.001	0.006
1.30–1.40	80.9 (7.97)	0.156	0.280	96.8 (5.57)	0.071	0.176
**B**		**Survival**			**CVDFS**	
**ABI**	**1-year**	**3-year**	**5-year**	**1-year**	**3-year**	**5-year**
0.80–0.89	81%	70%	41%	88%	80%	57%
0.90–0.99	89%	80%	44%	92%	88%	62%
1.00–1.09	90%	84%	65%	95%	91%	79%
1.10–1.19	92%	80%	60%	96%	92%	82%
1.20–1.29	93%	85%	67%	96%	93%	80%
1.30–1.40	85%	73%	69%	96%	87%	87%

ABI = ankle brachial index, CVDFS = cardiovascular death-free survival, *p* value log rank test, SE = standard error.

## Data Availability

The data are available upon request. Turku CRC turkucrc@tyks.fi.
